# Innovation in catheter design for intra-arterial liver cancer treatments results in favorable particle-fluid dynamics

**DOI:** 10.1186/s13046-015-0188-8

**Published:** 2015-08-01

**Authors:** Andor F. van den Hoven, Marnix G.E.H. Lam, Shaphan Jernigan, Maurice A.A.J. van den Bosch, Gregory D. Buckner

**Affiliations:** Department of Radiology and Nuclear Medicine, University Medical Center Utrecht, Room E.01.132, Heidelberglaan 100, 3584 CX Utrecht, The Netherlands; Department of Mechanical and Aerospace Engineering, North Carolina State University, 911 Oval Drive, Raleigh, North Carolina 27695 USA

**Keywords:** Radioembolization, Particle-fluid dynamics, Catheter design, Vascular model, Liver tumors

## Abstract

**Background:**

Liver tumors are increasingly treated with radioembolization. Here, we present first evidence of catheter design effect on particle-fluid dynamics and downstream branch targeting during microsphere administrations.

**Materials and methods:**

A total of 7 experiments were performed in a bench-top model of the hepatic arterial vasculature with recreated hemodynamics. Fluorescent microspheres and clinically used holmium microspheres were administered with a standard microcatheter (SMC) and an anti-reflux catheter (ARC) positioned at the same level along the longitudinal vessel axis. Catheter-related particle flow dynamics were analyzed by reviewing video recordings of UV-light illuminated fluorescent microsphere administrations. Downstream branch distribution was analyzed by quantification of collected microspheres in separate filters for two first-order branches. Mean deviation from a perfectly homogenous distribution (DHD) was used to compare the distribution homogeneity between catheter types.

**Results:**

The SMC administrations demonstrated a random off-centered catheter position (in 71 % of experiments), and a laminar particle flow pattern with an inhomogeneous downstream branch distribution, dependent on catheter position and injection force. The ARC administrations demonstrated a fixed centro-luminal catheter position, and a turbulent particle flow pattern with a more consistent and homogenous downstream branch distribution. Quantitative analyses confirmed a significantly more homogeneous distribution with the ARC; the mean DHD was 40.85 % (IQR 22.76 %) for the SMC and 15.54 % (IQR 6.46 %) for the ARC (*p* = 0.047).

**Conclusion:**

Catheter type has a significant impact on microsphere administrations in an in-vitro hepatic arterial model. A within-patient randomized controlled trial has been initiated to investigate clinical catheter-related effects during radioembolization treatment.

**Electronic supplementary material:**

The online version of this article (doi:10.1186/s13046-015-0188-8) contains supplementary material, which is available to authorized users.

## Background

Primary liver tumors (hepatocellular carcinoma, intrahepatic cholangiocarcinoma) and liver metastases affect a great number of cancer patients worldwide, and advanced disease stages are generally associated with poor prognosis [1–4]. Only a minority of patients are eligible for potentially curative surgery, and the efficacy of palliative systemic therapy is dependent on tumor type [5–8]. In the last decade, image-guided treatment techniques have evolved as another therapeutic option, and minimally invasive, trans-catheter treatments such as transarterial chemoembolization and intra-arterial radioembolization have found their way to patients with irresectable and chemorefractory disease [9, 10].

During radioembolization treatment, radioactive microspheres are administered through a catheter placed in the hepatic arterial vasculature. Since liver tumors are almost exclusively vascularized by the hepatic artery, and healthy liver tissue receives the majority of its blood supply from the portal vein, arterial blood flow should preferentially transport the microspheres towards tumorous tissue, where they lodge in the distal vessels surrounding tumors and emit tumoricidal high-energy β-radiation, while relatively sparing healthy liver tissue [11]. Yet, individual tumors may receive sub-therapeutic doses of radioactivity as a result of the complex interplay between tumor vascularization, particle-fluid dynamics and catheter placement [12–14].

So far, the standard end-hole microcatheter (SMC) has been the undisputed device of choice for microsphere administrations. However, a novel catheter type has recently been developed specifically for trans-catheter liver cancer treatments. This anti-reflux catheter (Surefire Infusion System, Surefire Medical Inc., Westminster, Co, USA) features a dynamically expandable tip that prevents reflux of particles in reverse flow conditions, while preserving normal antegrade blood flow [15]. In addition, the catheter orifice is fixed in the center of the vessel lumen (centro-luminal position). These marked differences may affect fluid-particle dynamics during microsphere administrations, and have a significant impact on tumor targeting during radioembolization.

Remarkably, fluid-particle dynamics are still inadequately understood, and the potential effects of catheter design and position remain unknown. The aim of this study is to enhance our understanding of this complex interplay by comparison of microsphere administrations with an ARC and SMC in the controlled environment of an experimental vascular model. In addition, we elaborate on potential clinical benefits that may be associated with favorable catheter characteristics.

## Methods

### Experiments

Table 1 shows an overview of the methods for all experiments performed in this study. The microsphere type, catheter position, injection technique and vascular model varied between experiments to address specific questions and test both catheter types across a variety of situations (see the specific methods section for a more detailed description). These parameters were, however, kept identical between the SMC and ARC administrations within the same experiments to warrant a valid comparison of the catheter types. The main objective of experiments 1–3 with the fluorescent microspheres was to document the particle outflow pattern for qualitative analysis. For experiments 4–7 with the holmium microspheres, the main objective was to extend the quantitative data on the consistency and homogeneity of the down-stream branch distribution with clinically validated microspheres. Secondary objectives were to assess the effect of injection force on downstream branch targeting, and to evaluate random cross-sectional catheter positioning.

**Table 1:** Description of experiments

Experiment	Microspheres	Catheter type	Catheter position	Injection technique	Vascular model
1	Fluorescent	SMC	Distal PHA	Manual	1
		ARC			
2	Fluorescent	SMC	Proximal PHA	Automatic	1
		ARC			
3	Fluorescent	SMC	Proximal PHA	Automatic	1
		ARC			
4	Holmium	SMC	RHA, proximal to S4A	Automatic	2
		ARC			
5	Holmium	SMC	RHA, proximal to S4A	Automatic	2
		ARC			
6	Holmium	SMC	RHA, proximal to S4A	Automatic	2
		ARC			
7	Holmium	SMC	RHA, proximal to S4A	Automatic	2
		ARC			

This table gives an overview of the experiments performed. Abbreviations: SMC = standard microcatheter; ARC = anti-reflux catheter; PHA = proper hepatic artery; RHA = right hepatic artery; S4A = segment 4 artery

### In vitro hepatic arterial model

**Fig. 1:**
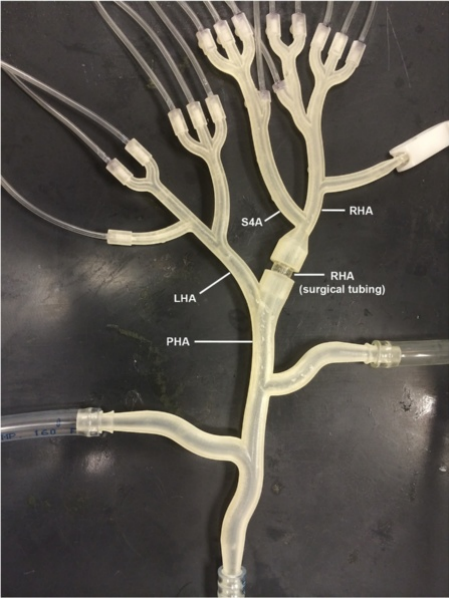
Photograph of the experimental hepatic arterial model (model #2). For practical reasons, the model is oriented upside down (different to orientation in a patient). Abbreviations: PHA = proper hepatic artery; LHA = left hepatic artery; S4A = segment 4 artery; RHA = right hepatic artery

**Fig. 2:**
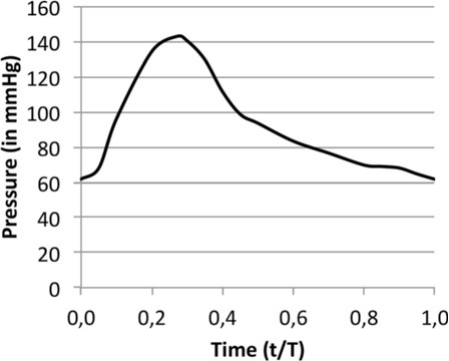
Recreated pressure profile of the hepatic arterial vasculature. The change in blood pressure (in mm Hg) is displayed for one cardiac cycle (per non-dimensional time unit with t = time point in cardiac cycle, and T = period of one cardiac cycle)

An in vitro hepatic arterial model was created to replicate hemodynamics and vessel geometry during microsphere administrations in the human hepatic arterial vasculature. The use of this model has been described before [16]. Central to this system was a rigid planar model fabricated by 3D printing. Two different models were used: a transparent model for the fluorescent microsphere administrations (experiments 1–3) to optimize the visibility of the microsphere flow, and a non-transparent model with a piece of surgical tubing inserted at the intended injection position to optimize vessel sealing by the ARC (experiments 4–7, Fig. 1). The geometry of both models was obtained from the branching pattern of three-dimensional CT imaging. The models consisted of a main bifurcation into left hepatic artery (LHA) and right hepatic artery (RHA), with the LHA terminating in six vessels (1.0 mm ID), and the RHA and segment 4 arterial (S4A) branch terminating in a total of ten vessels (1.0 mm ID). Hemodynamics was regulated by a closed-loop, dynamically pressurized system. At the proximal side of the vascular model, a fluid supply reservoir was connected to two parallel configured, computer-controlled pumps, a gear pump (Greylor Corporation, Cape Coral, FL) and a custom-made positive displacement pump that induced pulsatile pressurization of the hepatic arterial model to resemble the cardiac cycle. A blood pressure profile with a systolic/diastolic value of 140/60 mm Hg and 60 cycles/min was chosen as target (Fig. 2), based on previous simulations of the hepatic arterial blood flow [17]. One-way valves in the fluid lines near the pumps prevented backflow. The distal vessels drained into open collection reservoirs, mounted on USB-interfaced laboratory scales. Pumps connected to the collection reservoirs intermittently recirculated the fluid back to the fluid supply reservoir. Real time mass measurements were used to quantify the intra-vascular flow rates. All terminal vessels ran through pinch valves that were iteratively adjusted to keep the flow rates at target level and increase peripheral resistance. A target flow velocity of 10 ml/min for each vessel (total flow rate 160 ml/min) was chosen for all administrations. This choice was based on reported flow rates of the right hepatic artery with a range of 29–225 mL/min, constituting 60 % of the total hepatic arterial flow, yielding a range of 48.3-375.0 mL/min for the proper hepatic artery [18–20]. To replicate blood viscosity, with a reported value of 3.49 cP at systolic shear rates [21] (ex vivo measurements corrected for hematocrit level), a 25/75 % glycerin/water solution was used. Adequate fluid viscosity (3.48 ± 0.42 cP at a shear rate of 150 s-1) was confirmed by measurements taken with a HAAKE™ Viscotester™ 550 (ThermoFisher Scientific, Waltham, MA).

### Catheter positioning

**Fig. 3:**
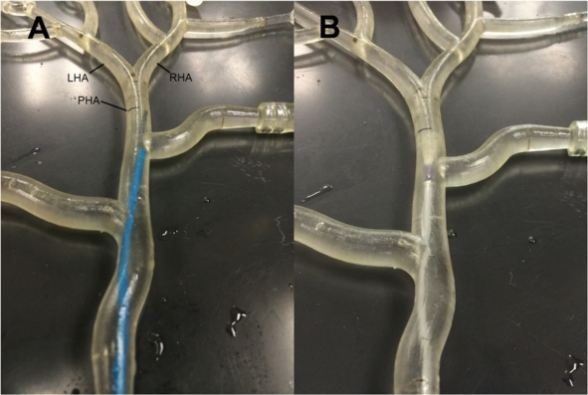
Catheter positions during fluorescent microsphere administrations. Photographs of the catheter positions in the geometry of the recreated hepatic arterial model, corresponding to the fluorescent microsphere administrations in Fig. 4. **a** Note the SMC tip deviation towards the right side. **b** Fixed centro-luminal position of the ARC. Abbreviations: LHA = left hepatic artery; RHA = right hepatic artery; PHA = proper hepatic artery. NB: the model is oriented up-side down

After achieving a constant flow velocity of 10 ml/min, the catheter was introduced into the model through a port proximal to the hepatic arterial model. The tip of the SMC (Progreat 2.7 Fr., Terumo Europe, Leuven, Belgium) and the ARC (Surefire Infusion System mT, Surefire Medical Inc., Westminster, Co, USA) were positioned at a target location on the longitudinal axis of the vessel. Different target locations were chosen to test both catheter types in various geometrical configurations. For the first experiment, this location was at 2 mm distance to the LHA/RHA bifurcation, for the second and third experiment a 5 mm distance was chosen (representative for a whole liver treatment with a proper hepatic artery injection). For the experiments with holmium microspheres in the second model (experiments 4–7), the catheters were positioned in the surgical tubing inserted in the RHA before the branching of the S4A (representative for a lobar treatment with a right hepatic artery injection). The position of the SMC in the cross-sectional vessel plane was a result of random placement, affected by the entire vessel geometry (Fig. 3a). Deviation of the SMC tip position was noted. The tip of the ARC was deployed just before the injection.

### Microsphere injections

A 200 mg dose of fluorescent red polyethylene microspheres (Cospheric LLC, Santa Barbara, Ca, USA) with a density of 1.20 g/cc and size of 27–32 μm was used for experiments 1–3. Fluorescent illumination of these microspheres is induced at a wavelength of 300–550 nm. 600 mg non-irradiated, ^165^Ho loaded poly (L-lactic acid) microspheres (QuiremSpheres, Quirem Medical Inc., Utrecht, The Netherlands) with a density of 1.40 g/cc and size of 30 μm (range 20–50 μm) were used for experiments 4–7. Both microsphere types were prepared for administration according to manufacturers instructions. The fluorescent microspheres were suspended in a tween surfactant solution (Tween 20) to prevent aggregation, and administered with 30 ml of water, whereas the holmium microspheres were suspended in an isotonic phosphate buffer, and administered with 30 ml of 0.9 % NaCl solution.

For the first experiment only, microspheres were injected by manual operation of a 30 cc syringe, using an intermittent short pulse pattern, similar to clinical practice. For a brief period of time, injection force was varied from minimal (pulse injection rate 0.3 ml/sec), to nominal (0.6 ml/sec), and ultimately excessive (1.2 ml/sec), in order to evaluate the impact of injection force on downstream branch distribution. The time points of different injection force were recorded. During the other experiments (experiments 2–7), an automatic syringe pump was used with standardized settings for injection (pulses of 0.5 ml, 1 sec interval between pulses, 5 ml/min) to eliminate all variability for an unbiased comparison between SMC and ARC administrations. Intra-vascular flow continued for 15 min after the beginning of administration.

### Qualitative analysis of catheter-related flow patterns

All fluorescent microsphere administrations were filmed using a mounted high definition camera, under UV-light illumination to assess catheter-related flow patterns qualitatively. The video recordings of all fluorescent microsphere administrations with the SMC and ARC were reviewed side-by-side to facilitate the comparison of particle flow patterns for each catheter type.

### Quantitative analysis of microsphere distribution

The distal vessels of the first-order branches (LHA/RHA for experiments 1–3, and S4A/RHA for experiments 4–7) drained into separate filters of Dutch-weave stainless steel with a rating of 10 μm (validated in-house for successful filtering of microspheres). These filters were weighed on a precision scale (AB135-S/FACT laboratory scale, Mettler-Toledo Inc., Columbus, Ohio, USA) prior to administration, and again post-administration after 12 h of heating at 190-250 °F in a digitally controlled Thermolyne 1500 furnace (ThermoFisher Scientific, Waltham, Mass., USA) to evaporate the residual water and glycerin. The measured change in mass was used to quantify the microspheres collected. The distribution over the main branches was subsequently determined by calculating the proportional weight change per filter as a function of the total weight change for both filters. In order to compare the distribution homogeneity across catheter types, the deviation from a perfect homogenous distribution (50:50 %), abbreviated as DHD, was calculated in percentage points (for example, a 10:90 % distribution results in a DHD of 40 %).

### Statistics

DHD was considered a continuous outcome variable. Data were summarized in median, range, and inter-quartile range (IQR) values (non-normally distributed). Differences in DHD between administrations with both catheter types were tested by means of a Wilcoxon signed-rank test for paired continuous data. Sample size calculation based on exploratory statistics after experiments 1–5 demonstrated that two additional experiments should be sufficient to demonstrate a statistically significant difference in DHD, with a power of 0.90 at an alpha-level of 0.05. A p-value < 0.05 was considered statistically significant. All statistics were performed in R version 3.1.1 for Mac OS X.

## Results

### Fluorescent microsphere administration demonstrates catheter-dependent flow pattern

**Fig. 4:**
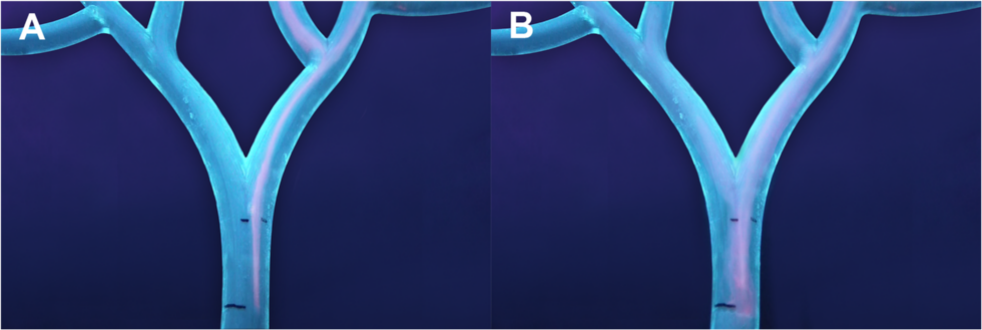
Catheter-related particle flow-dynamics. Composite figure of the fluorescent microsphere administrations with the SMC (**a**) and ARC (**b**) Images of fluorescent microsphere administrations were created by overlaying frames from a representative part of the experiment 3 video (Additional file 1: Movie S1), to show the typical catheter-related particle flow pattern. The background of images (**a**) and (**b**) was edited in Adobe Photoshop to emphasize an area of interest. **a** Off-centered SMC position, laminar outflow pattern and absence of microsphere flow towards the LHA. **b** Centro-luminal ARC position, turbulent outflow pattern (note the eddy current adjacent to the ARC tip) and more homogenous microsphere distribution over the LHA and RHA

A clear qualitative difference in particle outflow pattern between the two catheters was observed, consistently over all experiments (Fig. 4a-b, and Additional file 1: Movie S1). The SMC administrations showed an ordered, streamlined outflow pattern, consistent with laminar flow. In contrast, the ARC administrations had a chaotic, broad-based outflow pattern, consistent with turbulent flow.

### Injection force affects downstream branch targeting with a standard end-hole microcatheter

**Fig. 5:**
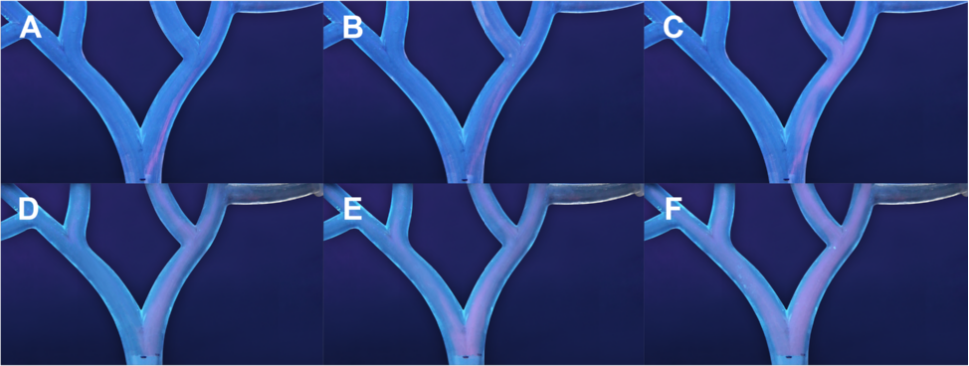
Effect of injection force on downstream branch targeting. This figure highlights the effect of injection force on downstream branch targeting. The images are constructed from the video recordings from experiment 1 (similar to Fig. 4). The upper row (**a**-**c**) displays the administrations with the SMC, and the lower row (**d**-**f**) displays the ARC administrations. Periods of minimal (**a**,**d**), nominal (**b**,**e**) and excessive (**c**,**f**) injection force were recorded. Injection force seems to have a more significant impact on the downstream distribution with the SMC than with the ARC. **a** SMC administration with minimal force only targets the right second-order branch. **b**-**c** Injection with nominal or excessive force also leads to targeting of the left second-order branch, but the LHA is not targeted at all. Note that the flow pattern seems to change from laminar to turbulent (**a**-**c**). **d** ARC administration with minimal force leads to preferential targeting of the RHA. However, microspheres are still visible in the second order branches of the LHA. **e**-**f** The ARC administration with nominal and excessive force leads to a homogenous distribution over all first and second-order branches

Random positioning of the SMC in the cross-sectional vessel plane resulted in slight deviations towards the right side. Review of the administration videos revealed that the LHA received minimal dosage with the SMC, and the second-order branch targeting differed remarkably when using minimal, nominal or excessive injection force (Fig. 5a-c). The ARC administrations showed a more homogeneous first-order branch distribution, but the RHA was still preferentially targeted. No evident difference in second-order branch targeting was noted between periods of minimal, nominal and excessive applied injection force (Fig. 5d-f).

### Downstream branch targeting is more consistent and homogenous with an anti-reflux catheter

Random cross-sectional positioning of the standard microcatheter resulted in catheter tip deviations towards the right vessel wall during experiments 1–3, towards the left vessel wall during experiments 4 and 6, and a centered position during the experiments 5 and 7. The results of the quantitative analysis are summarized in Table 2. The downstream branch distribution was significantly more homogenous with the ARC than with the SMC (Wilcoxon signed-rank test, V = 26, *p* = 0.047). The median DHD was 40.85 % (range 8.13-50 %, IQR 22.76 %) for the SMC, and 15.54 % (range 0.55-32.45, IQR 6.46 %) for the ARC across all experiments. Furthermore, the homogeneity and consistency of the downstream distribution seemed dependent on cross-sectional catheter position for the SMC. Only the experiments with a centered cross-sectional SMC position (experiments 5 and 7) showed a relatively homogeneous distribution (DHD 8.13 % and 10.50 % respectively). The distribution was skewed (DHD range 39.68 % - 50 %) towards the catheter tip direction for experiments with an off-centered cross-sectional SMC position (experiments 1–4 and 6).

**Table 2:** Microsphere distribution

Experiment	Off-centered SMC position?	SMC administrations		ARC administrations		Deviation from homogeneous distribution	
		*LHA/S4A*	*RHA*	*LHA/S4A*	*RHA*	*SMC*	*ARC*
1	Yes (right side)	3,66 %	96,34 %	17,55 %	82,45 %	46,34 %	32,45 %
2	Yes (right side)	0,64 %	99,36 %	37,52 %	62,48 %	49,36 %	12,48 %
3	Yes (right side)	0 %	100 %	49,45 %	50,55 %	50,00 %	0,55 %
4	Yes (left side)	89,68 %	10,32 %	65,63 %	34,37 %	39,68 %	15,63 %
5	No	41,87 %	58,13 %	57,42 %	42,58 %	8,13 %	7,42 %
6	Yes (left side)	90,95 %	9,05 %	32,82 %	67,18 %	40,95 %	17,18 %
7	No	39,50 %	60,50 %	35,46 %	64,54 %	10,50 %	14,54 %

The microsphere distribution results are summarized in this table. The distribution over LHA or S4A and RHA are shown for both catheter types. The column LHA/S4A targeting refers to LHA for experiments 1–3, and S4A for experiments 4–7. The deviation from homogeneous distribution (DHD) is the distance to a perfect homogenous distribution (50 %-50 %) in percentage points. Abbreviations: LHA = left hepatic artery; S4A = segment 4 artery; RHA = right hepatic artery; SMC = standard microcatheter; ARC = anti-reflux catheter

## Discussion

In this report, we present the first evidence that a SMC and ARC differ substantially with regard to cross-sectional catheter position, particle outflow pattern, and downstream branch distribution during the administration of microspheres in an in vitro hepatic arterial model.

The use of a SMC has long been standard clinical practice for radioembolization procedures. This may be explained by its convenient use, and the fact that potential problems with random cross-sectional catheter position or inadequate downstream branch targeting are not easily visualized during angiographic procedures. In our study, administrations with an SMC exhibited a laminar particle flow pattern. Under normal conditions, arterial blood flow is laminar. This means that blood flows in streamlines parallel to the vessel wall, without lateral mixing [22]. The microspheres seemed to drift along these ordered streamlines. The downstream distribution therefore becomes dependent on random cross-sectional catheter positioning. The SMC induced a homogenous downstream branch distribution, only in the experiment in which it was reasonably centered. In the other 5/7 experiments, the SMC was off-centered, and a very heterogeneous downstream branch distribution was observed, with one of the targeted main branches receiving only 0-10 % of injected microspheres. Interestingly, qualitative video analysis of the first experiment also suggests that the downstream branch distribution may be dependent on injection force. We theorize that this is related to the difference between particle release velocity and blood flow velocity. Low force injection results in particle release velocities lower or equal to the local blood flow velocity. Therefore, microspheres follow the streamline into which they are released [17, 23–25]. On the contrary, high injection force results in a particle release velocity exceeding the local blood flow velocity, which allows microspheres to cross streamlines laterally.

In radioembolization treatment, it is important to achieve adequate coverage of the entire tumor-bearing liver tissue [26, 27]; therefore, a homogenous distribution over first-order branches is a minimum requirement. If the tumors are confined to a specific part of the liver, a more distal catheter position is often chosen [28], limiting the influence of flow on microsphere distribution. Furthermore, particle distribution is generally assumed to be consistent over repeated administrations with the catheter placed at the same position in the longitudinal axis of the vessel. In current practice, the administration of ^90^Y microspheres is simulated in the week(s) before treatment by injection of technetium-99 m macroaggregated albumin (^99m-^Tc-MAA) particles from the same longitudinal catheter position, to rule out extrahepatic microsphere deposition or significant liver-to-lung shunting [29]. Various studies demonstrated that the ^99m-^Tc-MAA-distribution does not accurately predict the post-treatment intrahepatic microsphere distribution [30–32]. Inconsistencies are often ascribed to marked differences in particle characteristics such as size, density, and embolic effect [33], but the results of our study suggest that random differences of the cross-sectional SMC position between procedures may also play an important role.

The ARC administrations revealed a turbulent particle flow pattern. The expandable tip likely breaks up the laminar columns in the antegrade flow; and once the flow has passed the tip, flow swirls (so called eddy currents) to create a chaotic, turbulent flow pattern into which microspheres are released. Combined with the fixed centro-luminal position of the catheter orifice, this leads to a more predictable and more homogenous microsphere distribution. This may have important implications for radioembolization.

For one, tumor targeting may be improved by using the ARC. The ultimate goal is to realize an adequate radioactive dose delivery to all tumors in the liver. Two studies demonstrated that inadequate treatment of at least one tumor occurs frequently in patients treated with radioembolization, which may explain some of the inconsistencies in reported tumor response rates [12, 13, 34]. Although tumor vascularization may complicate matters in practice, we expect that a more homogenous downstream branch targeting is paramount to achieve adequate tumor coverage, which should in turn translate in to improved treatment efficacy.

Furthermore, a more consistent microsphere distribution over repeated administrations may increase the predictability of the treatment effect. In this light, the combination of the self-centering ARC and holmium-166 (^166^Ho) microspheres is especially interesting. Holmium-166 microspheres have been developed and clinically validated as an alternative to ^90^Y microspheres for radioembolization, with the added value of γ-radiation based SPECT imaging, and the option to administer a scout dose of identical ^166^Ho microspheres before treatment instead of ^99m-^Tc-MAA [12, 35]. Administering identical particles during the scout dose procedure in the same longitudinal and axial catheter position as during treatment, may be the key to accurately predict the intrahepatic distribution of the therapeutic microspheres. An accurate prediction would enable tailoring of the treatment strategy towards individual patients. Current treatment activity calculations for ^90^Y microspheres are based on empirically determined thresholds for a maximum tolerable whole liver absorbed dose, assuming a perfectly homogenous microsphere distribution ratio, i.e. a tumor to non-tumor (T/N) ratio of 1 [14]. This worst-case scenario approach has been adopted, because it is currently not possible to accurately determine the individual T/N ratio prior to treatment. If the scout dose distribution truly reflects the post-treatment distribution, a minimal effective tumor dose can be targeted, while respecting a maximal tolerable healthy liver dose. Consequently, patients with a favorable T/N ratio on scout dose imaging may be treated more aggressively to enhance treatment efficacy, while a more careful approach could be selected for patients with an unfavorable T/N ratio to minimize toxicity.

Clinical studies on the use of the ARC have mainly focused on its anti-reflux characteristics, which obviates the need to coil embolize gastrointestinal branches during treatment workup, enabling a simpler and less time-consuming workflow [15, 36, 37]. In another study, it has been shown that the deployment of the ARC tip decreases blood pressure in the down-stream vascular compartment, but the consequences of this phenomenon remain uncertain [38]. Pasciak et al. recently performed the first clinical study in which down-stream particle distribution was compared between ARC and SMC administrations, using a within-patient controlled study design. This study suggested that the use of the ARC improves selective tumor targeting, with healthy liver uptake decreasing by 24 %-89 % and tumor uptake increasing by 33 %-90 % [39]. These results should, however, be interpreted with caution since ^99m^Tc-MAA was used to compare the intrahepatic particle distribution, instead of therapeutic ^90^Y-microspheres.

Disadvantages of the ARC include a more complex catheterization compared with the SMC, and the risk of vasospasm after deployment of the catheter tip. Therefore, interventional radiologists should familiarize themselves with the special technique that is required for its use. A vasoactive drug such as nitroglycerine can be used to prevent or remedy vasospasm [37].

The controlled environment of the experimental model enabled us to enhance our understanding of the interplay between catheter design/positioning, injection force, and fluid-particle dynamics through observation and quantitative experiments. Clinical hemodynamics were replicated as closely as possible by using pulsatile flow with a flow rate, pressure profile and fluid viscosity based on previously published in-vivo measurements, in a 3D printed vascular branching model. Nevertheless, it is impossible to match the complexity of clinical reality. Clear differences include a more complex three-dimensional geometry of the hepatic arterial vasculature, the presence of multiple tumors that affect blood flow, the elasticity and responsiveness of arterial blood vessels, and the potential occurrence of blood flow stasis during administration of microspheres. Some of these factors, such as preferential tumor vascularization, may potentially reduce the catheter-related differences that were observed. However, considering the fact that evident differences were observed in a model with a relatively simple geometry that should facilitate a homogenous down-stream branch distribution, we expect other factors to exaggerate these differences in clinical practice.

We have initiated a clinical trial to validate the findings of this study in vivo, and determine the catheter-related effects on patient outcome (clinical trials.gov identifier: NCT02208804). In short, we will perform a phase II trial in 25 patients with irresectable, chemorefractory, and liver-dominant colorectal liver metastases. All patients will receive a pretreatment procedure during which a scout dose of ^166^Ho microspheres will be administered, followed by a therapeutic procedure on the same day. The effects of both catheter types will be compared within subjects, by randomly allocating the ARC to the administrations in the left or right hepatic artery, and using the SMC on the other side. This approach is justified, since the left and right hemi-livers are functionally independent, and treatment effects are limited to those areas. The primary endpoint is the T/N ratio of the radioactivity concentration on post-treatment SPECT. Secondary endpoints include absorbed tumor dose and healthy liver tissue dose, tumor response, the predictive value of the scout dose distribution, infusion efficiency, dose–response relationship, clinical toxicity and overall survival. The sample size is based on an expected factor 1.25-fold difference in T/N ratio (deemed clinically relevant): a median T/N ratio 2.0 for the ARC administrations versus 1.6 for the SMC administrations.

## Conclusions

Using an ARC during microsphere administrations in a surrogate hepatic arterial model was associated with a favorable particle outflow pattern, a fixed centro-luminal catheter position, and a significantly more homogeneous downstream branch distribution, compared with the use of a SMC. These effects may have important implications for liver tumor treatments with radioembolization, which will be subject of investigation in a within-patient randomized controlled trial.

## Additional file

Additional file 1: Movie S1. Catheter-related particle flow dynamics: SMC versus ARC. This video fragment shows a part (first 35 s) of the fluorescent microsphere administration with the SMC (left side) and ARC (right side) in the third experiment.
